# Atypical chronic inflammatory demyelinating polyradiculoneuropathy: recent advances on classification, diagnosis, and pathogenesis

**DOI:** 10.1097/WCO.0000000000000979

**Published:** 2021-07-13

**Authors:** Pietro Emiliano Doneddu, Marta Dentoni, Eduardo Nobile-Orazio

**Affiliations:** a Neuromuscular Diseases and Neuroimmunology Service, IRCCS Humanitas Clinical and Research Institute, Rozzano; b Department of Medical Biotechnology and Translational Medicine, Milan University, Milan, Italy

**Keywords:** atypical chronic inflammatory demyelinating polyradiculoneuropathy, distal acquired demyelinating symmetric neuropathy, Lewis--Sumner syndrome, MADSAM

## Abstract

In recent years, there has been an intense debate in literature regarding the definition of the individual variants of chronic inflammatory demyelinating polyradiculoneuropathy (CIDP), their possible pathogenetic mechanisms, and impact in the diagnosis of CIDP.

**Recent findings:**

The 2021 European Federation of Neurological Societies/Peripheral Nerve Society (EFNS/PNS) guidelines revised the definition of the individual CIDP variants and implemented their diagnostic criteria. Diagnosis of atypical CIDP is challenging and misdiagnosis is frequent, leading to diagnostic delay and consequent greater accumulation of disability and treatment dependency. Recent studies suggest that patients with typical CIDP have an antibody-mediated mechanism of neuropathy whereas in those with Lewis--Sumner syndrome (LSS) neuropathy is preferentially mediated by macrophages and T cells.

**Summary:**

Although the validity of the 2021 EFNS/PNS diagnostic criteria for atypical CIDP is unknown, they will hopefully lead to greater uniformity in the selection of patients to be enrolled in future studies and to a greater diagnostic accuracy. New data are emerging on the possible pathological mechanisms of individual variants and this could result in the discovery of specific diagnostic biomarkers and new therapies.

## INTRODUCTION

Chronic inflammatory demyelinating polyradiculoneuropathy (CIDP) is a rare immune-mediated neuropathy with a very heterogeneous clinical presentation. Along with a typical clinical phenotype (typical CIDP), defined as symmetric sensorimotor neuropathy involving proximal and distal segments of the four limbs with a relapsing or progressive course of at least 2 months, a few atypical variants have been described (atypical CIDP) [1–6,7^▪▪^,^8^^▪▪^,^9^–^40^]. These variants include distal acquired symmetric demyelinating neuropathy (DADS), Lewis--Sumner syndrome (LSS), focal CIDP, pure motor and pure sensory CIDP. Recently a few other variants have been proposed by some authors, including chronic immune sensory polyradiculoneuropathy (CISP and CISP-plus), chronic immune motor polyradiculoneuropathy (CIMP), and chronic immune sensorimotor polyradiculoneuropathy (CISMP) [41,42^▪^,^43^,^44^,45^▪^]. It is still unclear whether the atypical CIDP variants should be considered different phenotypes of the same disease or clinical entities with a different pathogenetic mechanism. In the recent years, an intense debate around atypical CIDP has taken place, particularly around three key issues that we will try to summarize here: the clinical boundaries of the individual variants, their weight in the diagnostic difficulty of CIDP, and their clinicopathological peculiarities. 

**Box 1:**
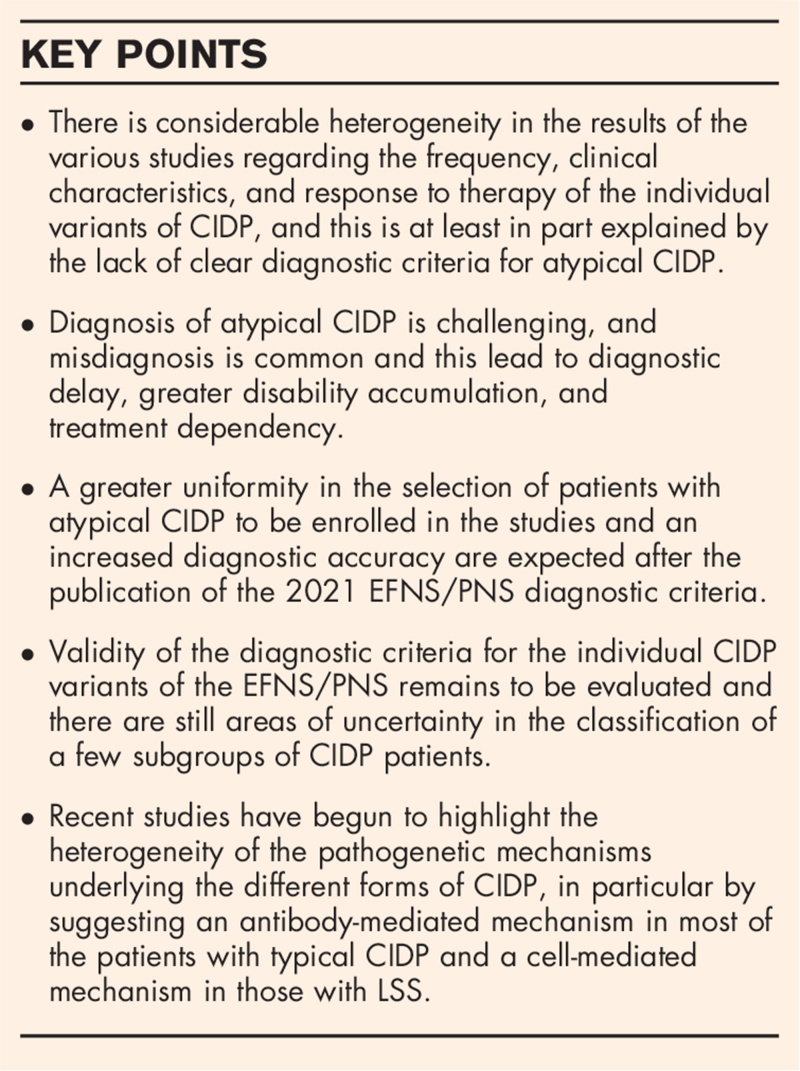
no caption available

## DEFINITIONS AND CLINICAL CHARACTERISTICS OF THE ATYPICAL CHRONIC INFLAMMATORY DEMYELINATING POLYRADICULONEUROPATHY VARIANTS

The 2010 European Federation of Neurological Societies/Peripheral Nerve Society (EFNS/PNS) guidelines for CIDP roughly defined the individual CIDP variants but did not provide criteria that allow to clearly establish the clinical boundaries of each of them [46]. The lack of universally recognized diagnostic criteria for atypical CIDP has favored over the years the proliferation of various definitions for the individual variants and this has resulted in a remarkable heterogeneity among studies regarding their reported frequency, clinical characteristics and response to therapy. Recently, an Italian study proposed a set of diagnostic criteria for atypical CIDP but its validity has not yet been demonstrated [7^▪▪^]. The application of these criteria in a large cohort of patients with atypical CIDP has surprisingly shown that 53% of the patients with atypical CIDP at onset progressed to typical CIDP during the course of the disease [7^▪▪^]. Progression to typical CIDP, however, was not absolute and a considerable proportion of patients maintained their atypical CIDP phenotype even after several years from symptoms onset [7^▪▪^]. Transition from atypical to typical CIDP has been questioned by some authors [47] but other studies confirmed that phenotypes can change over times [9,34,35]. Whether this progression reflects only a greater spread of neuropathy or is instead the result of specific pathogenetic mechanisms is still to be clarified.

### Distal acquired symmetric demyelinating neuropathy

In 2000, Katz *et al*. [1], described DADS as a distal, symmetric, sensory, or sensorimotor neuropathy sparing proximal limb, neck, and facial muscles. In their study, 60% of the patients had an IgM paraprotein and at least 33% of them were positive for anti-MAG (myelin-associated glycoprotein) antibodies [1]. Being an exclusion criterion for CIDP diagnosis, in the subsequent descriptions of DADS, only patients with negative anti-MAG antibodies were included, whereas idiopathic DADS was considered a variant of CIDP. Still, Larue *et al.*[3] found that 60% of the patients with DADS had a monoclonal gammopathy (40% IgG and 20% IgM). Association between DADS phenotype and IgM paraprotein was also recently confirmed in a study that has investigated the frequency and role of comorbidities in a large cohort of CIDP patients and found that 12.5% of the patients with DADS had an IgM monoclonal gammopathy (versus 5.5% of the patients with typical CIDP) [48^▪▪^]. According to some studies, DADS is the most common variant of CIDP, with a frequency that ranges from 2 to 15% (Table 1). Although DADS is defined as predominantly distal, the exact proximal to distal gradient of motor and sensory deficits was not specified neither in the 2010 EFNS/PNS guidelines nor in their revision [46,49^▪▪^].

**Table 1:** Reported diagnostic criteria, clinical characteristics and response to treatment for distal acquired demyelinating symmetric neuropathy

References	Number of patients or frequency	Clinical definition	Electrodiagnostic criteria for diagnosis	Exclusion criteria with respect to the specific variant	Disability (compared with typical CIDP)	Response to treatment
2000	30	Distal, symmetric, sensory or sensorimotor neuropathy sparing proximal limb, neck, and facial muscles [1]	NM	Proximal and distal weakness involving all four limbs or neck or facial muscles; motor symptoms dominating the clinical picture	NM	4/5 (80%) improved after any treatment
2009	6.5%	As per 2010 EFNS/PNS guidelines [2]	NM	NM	NM	NM
2011	6.8%	Clinical profile of DADS [3]	Disproportionately prolonged motor distal latencies (DL) resulting in a TLI 0.25 or less in at least two nerves	NM	NM	6/9 (67%) patients improved with IVIg, 2/2 (100%) with PLEx, and 1/2 (50%) with steroids
2013	2%	As per 2010 EFNS/PNS guidelines [4]	NM	NM	NM	NM
2014	3	As per 2010 EFNS/PNS guidelines [5]	NM	NM	Lower	3/3 (100%) patients improved after any treatment
2015	5%	As per 2010 EFNS/PNS guidelines [6]	NM	Clinical picture of typical CIDP or other atypical CIDP forms	NM	0/5 improved after any treatment
2019	7%	Symmetric, sensory or sensorimotor polyneuropathy starting distally in the LL, without proximal limb–trunk–face involvement (length-dependent fashion). Other possible symptoms include ataxia, neuropathic pain, cramps, fatigue, autonomic symptoms, tremor. UL distal sensory or sensorimotor symptoms and signs occurring later (at least after 1 year from onset) [7^▪▪^]	With or without increased distal motor latency	1. Cranial nerve involvement. 2. Proximal limbs, trunk, face involvement. 3. Weakness without sensory symptoms. 4. symptoms and signs starting in the UL	Lower	9/16 (56%) patients improved after steroids and 9/18 (50%) after IVIg
2019	15%	Distal-dominant, sensorimotor symmetric polyneuropathy [8^▪▪^]	NM	Clinical picture of typical CIDP or other atypical variants	Similar	NM
2020	13%	As per 2010 EFNS/PNS guidelines [9]	NM	Clinical picture of typical CIDP or other atypical variants.	Lower	NM
2020	6.8%	As per 2010 EFNS/PNS guidelines [10]	NM	Clinical picture of typical CIDP or other atypical variants.	Higher	NM

CIDP, chronic inflammatory demyelinating polyradiculoneuropathy; DADS, distal acquired demyelinating symmetric neuropathy; DL, distal latency; EFNS/PNS, European Federation of Neurological Societies/Peripheral Nerve Society; IVIg, intravenous immunoglobulin; LL, lower limbs; NM, not mentioned; PLEx, plasma exchange; TLI, terminal latency index; UL, upper limbs.

Response to treatment in DADS was initially reported to be similar to that of CIDP [1,3] but subsequent studies showed that this variant is likely to exhibit a lower response to therapy [6,7^▪▪^]. In larger cohorts, overall response to treatment and response to intravenous immunoglobulin (IVIg) in DADS was lower compared with typical CIDP [7^▪▪^]. Most studies report DADS as a mild form of CIDP [5,7^▪▪^,^9^].

### Lewis--Sumner syndrome

LSS was initially defined as a sensory or sensorimotor multineuropathy with persistent motor nerve conduction blocks [11–13]. According to some reports, this is the most common variant of CIDP (Table 2). Later, other authors have defined LSS as an asymmetric polyneuropathy [6,8^▪▪^,^15^–^18^,^20^,^23^], although only a few of them specified the degree of asymmetry necessary for diagnosis and how to quantify it [6,8^▪▪^,^23^]. Even the 2010 EFNS/PNS diagnostic criteria for CIDP defined LSS as an asymmetric polyneuropathy [46]. Then, other authors have eliminated the presence of conduction blocks as a diagnostic criterion [2,4,5,6,8^▪▪^,^9^,^10^,^14^–^16^,^19^,^21^,^22^]. In the Italian CIDP database, where LSS was defined as a multineuropathy, 37.5% of the patients with the typical form had an asymmetric but not multifocal CIDP including 9.5% with a slight asymmetry [one Medical Research Council (MRC) point difference between the two sides] [7^▪▪^]. These figures are interesting as they show that the asymmetric form of CIDP is much more frequent than expected from a clinical entity that is considered ‘atypical’. It cannot, however, be excluded that, as in vasculitic neuropathies, a certain number of patients with multineuropathic CIDP evolve over time towards an asymmetrical form. Finally, the 2021 EFNS/PNS criteria has defined LSS as a sensory or sensorimotor multineuropathy specifying that its clinical presentation is usually asymmetric [49^▪▪^].

**Table 2:** Reported diagnostic criteria, clinical characteristics and response to treatment for Lewis--Sumner syndrome

References	Number of patients or frequency	Clinical definition	Electrodiagnostic criteria for diagnosis	Exclusion criteria with respect to the specific variant	Disability (compared with typical CIDP)	Response to treatment
1982	5	Mononeuritis multiplex [11]	Multifocal persistent CB	NM	NM	2/2 (100%) patients improved after steroids
1997	16	Motor and sensory mononeuropathy multiplex [12]	Evidence of demyelination including CB	NM	NM	80% of patients improved after steroids
1999	11	Multifocal motor and sensory mononeuropathies [13]	CB or other features of demyelination	Symmetrical polyneuropathy	NM	5/9 (56%) patients improved after IVIg and 3/6 (50%) after steroids
1999	10	Symptoms and findings involved solely or predominantly the UL [14]	NM	Generalized, sensorimotor polyneuropathy or pure sensory and pure motor syndromes	Similar	5/9 (56%) patients improved after IVIg, 0/6 after steroids, PE, or cyclophosphamide improved
2000	6	Asymmetric sensory or sensorimotor polyneuropathy [15]	NM	NM	NM	6/6 (100%) improved after IVIg, 0/2 after steroids, 0/1after cyclophosphamide
2003	6%	Asymmetrical or multifocal motor sensory deficit [16]	NM	NM	NM	0/1 after steroids
2004	23	Asymmetrical sensory or sensorimotor neuropathy [17]	Persistent CB	Pure motor neuropathy or symmetrical polyneuropathy	NM	54% improved after IVIg and 33% after steroids
2005	13	Asymmetrical limb weakness at onset, and motor involvement in the distribution of at least two different peripheral nerves; objective clinical sensory involvement [18]	One site with definite CB or one site with probable CB in the UL, and at least one sensory action potential amplitude less than 80% of the lower limit of normal	NM	NM	2/8 (25%) improved after steroids, 8/13 (62%) after IVIg, 1/4 after PLEx
2009	8	Progressive focal or asymmetric sensory or sensorimotor neuropathy in the UL [19]	NM	Pure persistent motor involvement	NM	7/8 (87%) patients improved after IVIg, 1/3 (33%) after steroids
2009	15%	As per 2010 EFNS/PNS guidelines [2]	NM	NM	NM	NM
2011	15	Asymmetric multifocal sensory or sensorimotor neuropathy with involvement of at least two different peripheral nerves [20]	At least two sites with definite or probable CB; reduced sensory nerve action potential amplitude in at least one nerve	Motor neuropathy or sensorimotor polyneuropathy	NM	7/15 (47%) patients improved after IVIg, 1/4 (25%) after steroids, 0/4 after PLEx
2014	8%	As per 2010 EFNS/PNS guidelines [4]	NM	NM	NM	NM
2014	10	As per 2010 EFNS/PNS guidelines [5]	NM	NM	Lower	8/10 (80%) patients improved after any treatment
2015	34%	Mononeuropathy multiplex or asymmetry of symptoms, determined as differences in muscle strength by one or more MRC scales in the homonymous muscles [6]	NM	Clinical picture of typical CIDP or other atypical variants	Lower	6/16 (38%) improved after IVIg, 1/6 (17%) after PLEx, 21/29 (72%) after steroids
2019	4%	Sensory symptoms, with or without weakness, in a multifocal distribution (unilateral focal CIDP included); Symptoms may start anywhere in the body. Other possible symptoms: cramps, fatigue, autonomic symptoms, ataxia, neuropathic pain, motor and/or sensory cranial nerve palsy [7^▪▪^]	With or without motor CB	1. Weakness in isolation, without sensory symptoms. 2. Symptoms/signs in a polyneuropathic distribution	Lower	6/9 (67%) patients improved after steroids, 5/12 (42%) after IVIg
2019	9	As per 2010 EFNS/PNS guidelines [21]	NM	NM	NM	7/7 (100%) improved after IVIg, 4/4 (100%) after steroids
2019	14%	Asymmetric sensorimotor neuropathy, featuring differences in muscle strength by one or more grades on the MRC scale in the bilateral muscles [8^▪▪^]	NM	Clinical picture of typical CIDP or other atypical variants	Similar	NM
2019	34	Chronic asymmetric sensorimotor multifocal neuropathy [22]	NM	NM	NM	3/10 (30%) improved after steroids, 14/15 after IVIg
2020	45	Sensory or sensorimotor mononeuropathy multiplex or asymmetric polyneuropathy defined as differences in muscle strength of one or more MRC points in the homonymous muscles or initial focal involvement if extension to another territory was observed during follow-up or pure motor onset if a predominantly sensory impairment occurred subsequently [23]	At least one CB	NM	Lower	23/35 (66%) patients improved after IVIg, 2/9 after steroids
2020	11.1%	As per 2010 EFNS/PNS guidelines [9]	NM	Clinical picture of typical CIDP or other atypical variants	Lower	NM
2020	23.5%	Mononeuropathy multiplex or asymmetric weakness with one or more MRC scale differences in the homonymous muscles [10]	NM	Clinical picture of typical CIDP or other atypical variants	Lower	NM
2021	7.9%	As per Doneddu *et al.*[24]	.	.	NM	60% refractory to first-line treatments with IVIg, steroids or PLEx

CB, conduction blocks; CIDP, chronic inflammatory demyelinating polyradiculoneuropathy; EFNS/PNS, European Federation of Neurological Societies/Peripheral Nerve Society; IVIg, intravenous immunoglobulin; LL, lower limbs; LSS, Lewis--Sumner syndrome; MRC, Medical Research Council; NM, not mentioned; PLEx, plasma exchange; UL, upper limbs.

In 1996, Thomas *et al.*[25] described a form of CIDP restricted to one or two upper limbs and labelled this form ‘focal CIDP’. Later, the 2010 EFNS/PNS CIDP guidelines included focal CIDP in the list of atypical CIDP as one of its variants [46]. There is, however, no clear evidence from the literature that this form is distinct from LSS and should be kept separate from it. Indeed, three of the nine patients originally described by Thomas *et al.*[25] had a neuropathy diffused in both upper limbs or lower limbs in a multineuropathic fashion whereas other authors included patients with a CIDP restricted to one limb under LSS [19]. There is also no evidence that focal CIDP has a different response to therapy compared with typical CIDP or LSS (Table 3). In the 2021 EFNS/PNS CIDP diagnostic criteria, focal CIDP has been included under LSS [49^▪▪^]. The reported response to therapy and particularly to high-dose IVIg in LSS vary among studies, although in most of them is reported to be unsatisfactory [6,7^▪▪^,^20^,^24^]. Some authors reported a reduced response to steroids [14,18,20,24]. Disability in patients with LSS is generally lower than that of patients with typical CIDP [5,6,7^▪▪^,^9^,^10^,^23^].

**Table 3:** Reported diagnostic criteria, clinical characteristics and response to treatment for focal chronic inflammatory demyelinating polyradiculoneuropathy

References	Number of patients or frequency	Clinical definition	Electrodiagnostic criteria for diagnosis	Exclusion criteria with respect to the specific variant	Disability (compared with typical CIDP)	Response to treatment
1996	9	Focal UL demyelinating neuropathy [25]	NM	NM	NM	3/5 (60%) patients improved after steroids, 6/6 (100%) after IVIg
2000	1	UL sensorimotor deficit [15]	CB	NM	NM	Improved after IVIg
2013	1	As per 2010 EFNS/PNS guidelines [26]	NM	NM	NM	Improved after IVIg
2019	1%	Included under the definition of LSS [7^▪▪^]	With or without motor CB	1. Weakness in isolation, without sensory symptoms. 2. Symptoms/signs in a polyneuropathic distribution	NM	NM
2019	1%	Motor or sensorimotor neuropathy confined to one limb [8^▪▪^]	NM	Clinical picture of typical CIDP or other atypical variants	NM	NM
2020	1	As per 2010 EFNS/PNS guidelines [27]	NM	NM	NM	Improved after IVIg and steroids in combination
2021	1	As per 2010 EFNS/PNS guidelines [28]	NM	NM	NM	Improved after IVIg
2021	30	Sensory or motor or sensorimotor neuropathy involving the brachial or lumbosacral plexus, or one or more peripheral nerves in one UL or one LL (monomelic distribution) [29]	NM	NM	NM	16/19 (84%) improved after IVIg, 0/5 after steroids

CB, conduction blocks; CIDP, chronic inflammatory demyelinating polyradiculoneuropathy; EFNS/PNS, European Federation of Neurological Societies/Peripheral Nerve Society; IVIg, intravenous immunoglobulin; LL, lower limbs; LSS, Lewis-Sumner syndrome; NM, not mentioned; UL, upper limbs.

### Pure motor chronic inflammatory demyelinating polyneuropathy

Pure motor CIDP was initially defined as a pure motor symmetric polyneuropathy [30,31]. Subsequently, some authors have included in its definition the electrophysiological criterion of normal sensory nerve conduction studies whereas others have admitted the presence of mild sensory symptoms [32,37]. As for pure sensory CIDP, the 2021 EFNS/PNS guidelines has now subclassified pure motor CIDP in a subform with normal sensory nerve conduction studies (‘pure motor CIDP’) and in another with abnormal sensory nerve conduction studies (‘motor-predominant CIDP’) [49^▪▪^].

Some of the initial reports of this clinical entity reported unresponsiveness or worsening with steroids while having an excellent response to IVIg [16,31,32] (Table 4). This early reports led to the 2010 EFNS/PNS guidelines in recommending IVIg as the initial treatment in pure motor CIDP [46]. A few subsequent studies have, however, not confirmed this early finding [7^▪▪^,^33^]. In a large Italian study, 43% of the patients with pure motor CIDP responded to steroids (versus 51% of typical CIDP patients) [7^▪▪^], whereas another study reported a response of 80% [33]. It has emerged from the data of the Italian CIDP database that none of the pure motor CIDP patients with normal sensory nerve conduction studies improved with steroid therapy whereas all improved patients had abnormal sensory conduction studies [7^▪▪^]. This finding, which was later confirmed by others [33], suggest that the electrophysiological involvement of sensory fibers is a marker of good response to steroids. It is possible, although speculative, that at least some of the patients with normal sensory electrophysiological studies have multifocal motor neuropathy, which typically is steroid-resistant. The 2021 EFNS/PNS CIDP guidelines has, however, confirmed the recommendation to consider IVIg as the initial therapy for pure motor CIDP [49^▪▪^].

**Table 4:** Reported diagnostic criteria, clinical characteristics, and response to treatment for pure motor chronic inflammatory demyelinating polyradiculoneuropathy

References	Number of patients or frequency	Clinical definition	Electrodiagnostic criteria for diagnosis	Exclusion criteria with respect to the specific variant	Disability (compared with typical CIDP)	Response to treatment
1996	3%	Motor symptoms and signs only [30]	NM	NM	NM	NM
1997	10%	Pure or predominantly motor syndrome [37]	NM	NM	NM	NM
2001	4	Pure motor involvement without sensory signs and symptoms [31]	Normal findings on electrophysiological testing of sensory fibres	NM	NM	4/4 (100%) patients improved after IVIg, 0/4 after steroids
2003	6%	Symmetrical pure motor deficit, with no sensory signs or symptoms [16]	Absence of sensory abnormalities on neurophysiological examination	NM	NM	20% of the patients improved after steroids, 5/5 (100%) after IVIg, 1/3 (33%) after PLEx
2009	2.2%	As per 2010 EFNS/PNS guidelines [2]	NM	NM	NM	NM
2010	5	Motor symptoms without sensory symptoms, except for mild distal paresthesia [32]	Almost normal results in sensory conduction studies	NM	NM	5/5 (100%) patients improved after IVIg, 2/2 (100%) after PLEx, 0/5 after steroids
2014	4%	As per 2010 EFNS/PNS guidelines [4]	NM	NM	NM	NM
2019	4%	1. Weakness, without sensory symptoms or signs, in a polyneuropathic distribution, symmetric or asymmetric. 2. Symptoms may start anywhere in the body. Other possible symptoms: cramps, fatigue, tremor, motor cranial nerve palsy [7^▪▪^]	With or without abnormal sensory nerve conduction studies	1. Sensory symptoms/signs including sensory ataxia. 2. Autonomic dysfunction. 3. Neuropathic pain. 4. Multifocal distribution	Similar	3/7 (43%) patients improved after steroids, 14/17 (82%) after IVIg
2019	4%	Motor neuropathy without sensory disturbance [8^▪▪^]	NM	Clinical picture of typical CIDP or other atypical variants	Similar	NM
2020	2.2%	As per 2010 EFNS/PNS guidelines [9]	NM	Clinical picture of typical CIDP or other atypical variants	NM	NM
2020	2%	Symmetric or asymmetric pure motor polyneuropathy without sensory symptoms or signs at diagnosis [33]	NM	Clinical picture of typical CIDP or other atypical variants	NM	12/16 (75%) patients improved after IVIg, 4/5 (80%) after steroids, 2/5 (40%) after PLEx

CIDP, chronic inflammatory demyelinating polyradiculoneuropathy; EFNS/PNS, European Federation of Neurological Societies/Peripheral Nerve Society; IVIg, intravenous immunoglobulin; NM, not mentioned; PLEx, plasma exchange.

### Pure sensory chronic inflammatory demyelinating polyneuropathy

The clinical boundaries between pure sensory CIDP and sensory DADS are not well clear, and this may possibly explain why in some studies, patients with a pure sensory neuropathy with a ‘stocking-and-glove distribution’ are included under DADS while in others under pure sensory CIDP [1,34,50]. This confusion probably underlies the large variability in the reported frequency of pure sensory CIDP among studies (1-24%) (Table 5). An Italian study proposed criteria for atypical CIDP in which sensory DADS was defined as a length-dependent neuropathy whereas sensory CIDP as a nonlength-dependent [7^▪▪^]. In this study, patients with sensory DADS but not those with pure sensory CIDP had a lower response to treatment compared with typical CIDP [7^▪▪^]. This figure has not yet been confirmed by other studies.

**Table 5:** Reported diagnostic criteria, clinical characteristics, and response to treatment for pure sensory chronic inflammatory demyelinating polyradiculoneuropathy

References	Number of patients or frequency	Clinical definition	Electrodiagnostic criteria for diagnosis	Exclusion criteria with respect to the specific variant	Disability (compared to typical CIDP)	Response to treatment
1992	10	Pure sensory peripheral neuropathy [34]	NM	Sensorimotor neuropathy even with sensory predominance	NM	3/5 (60%) patients improved after steroids, 2/5 (40%) after PLEx
1995	5	Pure sensory symptoms and signs [35]	NM	NM	NM	One patient improved after IVIg but then required steroids; another patient improved after PLEx but then required IVIg; a third patient improved after IVIg
1996	11%	Sensory symptoms and signs only [30]	NM	NM	NM	NM
1996	13.6%	Pure sensory neuropathy [36]	NM	NM	NM	3/3 (100%) patients improved after IVIg
1997	12%	Predominantly sensory syndrome with normal or virtually normal strength [37]	NM	NM	NM	NM
1999	7	Pure sensory neuropathy [38]	NM	NM	NM	3/4 (75%) patients improved after IVIg, 1/1 after steroids
1999	6%	Mild sensory symptoms only [39]	NM	NM	NM	NM
2003	5%	Sensory deficit in absence of muscle weakness [16]	NM	NM	NM	2/4 (50%) patients improved after steroids, 1/1 after IVIg
2004	8	Chronic sensory polyneuropathy [40]	Nondiagnostic electrophysiological studies with diagnostic sural nerve biopsies	NM	NM	4/8 (50%) patients improved after IVIg
2009	23.9%	As per 2010 EFNS/PNS guidelines [2]	NM	NM	NM	NM
2014	4%	As per 2010 EFNS/PNS guidelines [4]	NM	NM	NM	NM
2015	1%	As per 2010 EFNS/PNS guidelines [6]	NM	Clinical picture of typical CIDP or other atypical variants	NM	NM
2019	3.5%	1. Sensory symptoms (including ataxia), without weakness, in a polyneuropathic distribution, symmetric or asymmetric. 2. Symptoms may start anywhere in the body excluding a length-dependent pattern (included under DADS). Other possible symptoms: neuropathic pain, fatigue, tremor, facial sensory symptoms [7^▪▪^]	With or without abnormal motor nerve conduction studies	1. Motor symptoms/signs including cramps and motor cranial nerve palsy. 2. Multifocal distribution. 3. Autonomic dysfunction	Similar	4/6 (67%) patients improved after steroids, 6/7 (86%) after IVIg
2019	14%	Pure sensory neuropathy without motor symptoms [8^▪▪^]	NM	Clinical picture of typical CIDP or other atypical variants	Lower	NM

CIDP, chronic inflammatory demyelinating polyradiculoneuropathy; DADS, distal acquired demyelinating symmetric neuropathy; EFNS/PNS, European Federation of Neurological Societies/Peripheral Nerve Society; IVIg, intravenous immunoglobulin; NM, not mentioned; PLEx, plasma exchange.

Although defined as a pure sensory neuropathy, most of the pure sensory CIDP cases so far described had subclinical electrophysiological involvement of the motor fibers but this is likely to be explained by the fact that signs of demyelination in the motor nerves were required by the 2010 EFNS/PNS diagnostic criteria for the diagnosis of CIDP [46]. Some descriptions of pure sensory CIDP without electrophysiological involvement of motor fibers have, however, been made [51]. In order to provide greater clarity, the 2021 EFNS/PNS criteria has now subclassified pure sensory CIDP in a subform with normal motor nerve conduction studies (‘pure sensory CIDP’) and in another with abnormal motor nerve conduction studies (‘sensory-predominant CIDP’) [49^▪▪^]. No studies have yet compared the clinical and immunological characteristics of these two CIDP subforms.

Response to treatment in pure sensory CIDP is reported to be similar to that of typical CIDP [7^▪▪^,^34^,^36^,^39^], whereas severity of the disease is reported to be similar or lower [7^▪▪^,^8^^▪▪^].

### Chronic immune sensory polyradiculoneuropathy, chronic immune sensory polyradiculoneuropathy-plus, chronic immune motor polyradiculoneuropathy, and chronic immune-mediated sensorimotor polyradiculopathy

CISP is generally considered pure sensory CIDP because of its similar clinical presentation characterized by only sensory symptoms without weakness [41]. Its peculiar feature is the selective involvement of the preganglionic root as evidenced by normal sensory nerve conduction studies, increased CSF protein levels, and thickened spinal roots at MRI [41]. It is a rare CIDP variant; in an Italian study on 460 CIDP patients, its frequency was 0.5% [7^▪▪^]. In the first description of 15 patients, all the patients had ataxia, nine had frequent falls, and six were severely disabled [41]. All of the treated patients had a rapid improvement, but relapsed on attempted tapering [41]. CISP-plus is a recently described variant in whom the disease extends beyond dorsal roots to also involve motor and postganglionic sensory nerve fibers, resulting in mild distal weakness and mild abnormalities on nerve conduction studies [42^▪^]. Its symptoms and response to therapy seem very similar to those of CISP [42^▪^]. CIMP is a chronic pure motor polyradiculopathy affecting the lumbosacral segments and sparing sensory, bowel, and bladder functions [43]. Imaging demonstrates nerve root enlargement of the cauda equina, and CSF protein are elevated [43]. To our knowledge, only one patient with CIMP has been reported so far. Eleven patients with an immune-mediated sensorimotor polyradiculopathy (CISMP) have been reported by two different reports [44,45^▪^]. Electrophysiological studies were normal in all these patients and a good response to treatment was observed in most of them [44,45^▪^]. Although all these rare forms were proposed by the authors as being part of the CIDP spectrum, the 2021 EFNS/PNS criteria mentioned only CISP and specified that it cannot still be considered as CIDP as there is not enough evidence to determine if it is demyelinating or related to sensory CIDP [49^▪▪^].

## DIAGNOSIS OF ATYPICAL CHRONIC INFLAMMATORY DEMYELINATING POLYRADICULONEUROPATHY

As for typical CIDP, no diagnostic biomarker exist for atypical CIDP making clinical and electrophysiological criteria essential for diagnosis. The 2021 EFNS/PNS criteria refined the diagnostic criteria for the CIDP variants and expanded the 2010 EFNS/PNS criteria by including sensory nerve conduction studies as a mandatory diagnostic criterion and by defining specific clinical and electrophysiological criteria for each CIDP variant [49^▪▪^]. Validity of these diagnostic criteria remains, however, to be established. Furthermore, these criteria still leave some areas of uncertainty. For instance, it is not clear how to classify patients with asymmetric but not multineuropathic CIDP or patients with a sensorimotor polyneuropathic CIDP only involving the proximal and distal segments of the lower limbs.

Atypical CIDP is a challenging diagnosis and its diagnostic workflow and differential diagnosis may differ compared with typical CIDP [52^▪^]. In one series of misdiagnosed patients, 44% of the patients misdiagnosed as CIDP were found to satisfy the EFNS/PNS criteria but they were all classified as ‘atypical’ [53]. Compared with the patients with typical CIDP, those with atypical CIDP more frequently were diagnosed in a university hospital and have a diagnostic delay [53,54^▪^]. This lead to a greater disability and more frequent fatigue and treatment dependency [54^▪^]. Patients with atypical CIDP phenotypes are also more likely to be falsely labelled as having CIDP (overdiagnosis) [52^▪^,^55^]. The reasons that may explain this diagnostic difficulty include the scarce adherence to the EFNS/PNS criteria, the inability to recognize the distinctive clinical and electrophysiological signs of CIDP and the clinical parameters indicative of a true response to therapy [52^▪^,^53^,^55^]. Given the complexity of the disease and its rarity, several authors have proposed that patients with an atypical CIDP phenotype or with an unexpectedly poor treatment response should be referred to CIDP expertise centres [52^▪^,^55^]. The 2021 EFNS/PNS guidelines have improved guidance regarding the diagnosis of CIDP in general and the specific diagnosis of the individual variants by suggesting a list of other conditions to be considered in the differential diagnosis and a series of diagnostic tests to be performed to exclude other causes [49^▪▪^].

## CLINICOPATHOLOGICAL CHARACTERISTICS OF THE CHRONIC INFLAMMATORY DEMYELINATING POLYRADICULONEUROPATHY VARIANTS

Although the cause of CIDP and its pathogenesis are still unknown, in the last years some progress has been made in deciphering the pathogenetic mechanisms underlying the disease. Several recent lines of evidence suggest that typical CIDP and its variants potentially have heterogeneous pathogenetic mechanisms.

Electrophysiological studies have shown that the distribution of lesions in the peripheral nervous system is different among the individual CIDP forms [6,8^▪▪^,^10^,56^▪^]. In typical CIDP, the most conspicuous electrophysiological alterations are the elongation of the F waves and the increase of distal motor latencies, whereas motor conduction blocks are more frequent in LSS [6,8^▪▪^,^10^,56^▪^]. These findings suggest that typical CIDP exhibits preferential involvement of the proximal and distal nerve segments whereas lesions in the middle nerve trunks are more common in LSS [6,8^▪▪^,^10^,56^▪^]. This might also explain why in LSS, the increase in CSF protein levels, which indicates the presence of lesions at proximal nerve segments, is less frequent and conspicuous. In support of this view, different magnetic resonance and ultrasound studies demonstrated hypertrophy predominantly in the nerve roots in patients with typical CIDP and patchy swelling of the nerve trunk in patients with LSS [57]. Similar findings have emerged from recent sural nerve biopsy studies, which showed the presence of uniform alterations with relative preservation of myelinated fibers and few axonal sprouts and onion-bulb formation in patients with typical CIDP, and instead, focal signs of demyelination with marked variation in the density of myelinated fibers among fascicles and conspicuous axonal sprouts in those with LSS [8^▪▪^]. The hypothesis, raised by some authors on the basis of these findings, is that in typical CIDP, the damage occurs in the proximal and distal portions of the nerve where the blood--nerve barrier is most deficient, and therefore, is likely mediated mainly by antibodies and humoral factors [6,8^▪▪^,^10^,56^▪^]. Notably, the 2021 EFNS/PNS CIDP guidelines proposed not to regard patients with antibodies against nodal--paranodal cell-adhesion molecules as CIDP variants as they have distinct clinical features, no overt inflammation or macrophage-mediated demyelination and do poorly respond to CIDP treatment, IVIg, in particular [5]. On the other hand, in LSS, the damage is likely mediated by T cells and macrophages that attack focal portions of the nerve with blood--nerve barrier breakdown [6,8^▪▪^,^10^,56^▪^]. This hypothesis may also explain the different response to treatment and outcome of LSS compared with typical CIDP. A recent study highlighted the presence of different underlying immunological mechanisms in typical CIDP and LSS [58^▪^]. DADS and sensory CIDP seem to be more heterogeneous, with some patients having findings similar to typical CIDP and others more similar to LSS [6,8^▪▪^,^10^,56^▪^]. It should be also underlined that, even among patients with typical CIDP there are some patients with electrophysiological and pathologic findings characteristic of LSS and vice versa, suggesting a higher level of pathogenetic complexity and the presence of overlapping mechanisms [6,8^▪▪^,^10^,56^▪^].

## CONCLUSION

Although the validity of the 2021 EFNS/PNS criteria for CIDP has not yet been assessed, they will lead to greater uniformity in the selection of patients to be enrolled in future studies and this, hopefully, will result in greater comparability of studies. Their implementation should also lead to an improvement in diagnostic accuracy. There are still, however, areas of uncertainty in the definition of the individual variants. Recent studies are starting to bring to light the pathogenetic mechanisms of the individual CIDP variants and this could result in the discovery of specific diagnostic biomarkers and new therapies.

## Acknowledgements

*None.*

### Financial support and sponsorship

*None.*

### Conflicts of interest

*E.N.-O. reports personal fees for Advisory or Scientific Board from Argenx, Belgium, Baxter/Takeda, Italy and USA, CSL-Behring, Italy and Switzerland Janssen, USA, Kedrion, Italy, Novartis, Switzerland, Roche, Switzeland, Sanofi, USA, outside the submitted work and travel grants to attend Scientific Meeting from Baxter, Grifols, Kedrion, and Novartis, Italy. P.E.D. has received travel grants to attend scientific meetings from CSL Behring and Kedrion. M.D. declared no conflicts of interest.*

## REFERENCES AND RECOMMENDED READING

Papers of particular interest, published within the annual period of review, have been highlighted as:

▪ of special interest
▪▪ of outstanding interest
